# ASTHMA AND COPD EXACERBATIONS: ED VISITS AND HOSPITALIZATIONS AFTER WILDFIRE SMOKE EXPOSURE AMONG OLDER ADULTS

**DOI:** 10.1093/geroni/igae098.2319

**Published:** 2024-12-31

**Authors:** Zhao Xiang Lin, Tal Sharon, Anita Szerszen

**Affiliations:** Northwell Health, New York, New York, United States; Staten Island University Hospital, New York, New York, United States; Northwell Health, New York, New York, United States

## Abstract

Global temperatures are rising, leading to more intense wildfires, as seen in Quebec, Canada, on June 2023, where 13 million acres burned. The resulting particulate matter may severely degrade air quality, posing risks to older adults (OAs) with respiratory diseases. Studies indicate Black/African American communities may be disproportionately affected by air pollution compared to white communities. This study evaluated the impact of wildfires on emergency department (ED) visits and hospitalizations for COPD and asthma among adults 65 and older. The observational cohort study assessed 1321 patient records from two representative New York City hospitals. Findings revealed no significant change in the overall volume of ED visits, hospitalizations, and median length of stay during the smoke-affected months compared to the previous year. However, there was a significant increase in non-white OAs’ ED visits and hospitalizations due to COPD or asthma: 27.19% in 2022 to 35.87% in 2023 (p = 0.0007), and more non-white patients with Medicaid insurance (80% in 2023, 71.43% in 2022). Significantly, more Black/African Americans required acute care: 16.74%, from 11.06% in 2022 (p = 0.003). These results indicate that worsening air quality may disproportionally impact non-white OAs. Black/African Americans and those with Medicaid insurance may more likely experience COPD or asthma exacerbation, requiring ED evaluation and/or hospitalization due to smoke pollution. The escalation in global temperatures and resulting wildfires may particularly affect OAs of color, especially those facing socioeconomic disadvantages. The findings underscore the importance of targeted public health strategies to address these disparities.

